# Dynamics of archaeal diversity and functionality in the piglet gut microbiome under common antimicrobial treatments

**DOI:** 10.3389/fcimb.2026.1833734

**Published:** 2026-07-16

**Authors:** Judith Guitart-Matas, Marc Bravo, Carla Tort-Miró, Noemí Giler-Baquerizo, Lorenzo Fraile, Yuliaxis Caldas-Ramayo, Maria Ballester, Lourdes Migura-Garcia

**Affiliations:** 1 Joint Research Unit Institut de Recerca i Tecnologia Agroalimentàries (IRTA)-Universitat Autònoma de Barcelona (UAB) in Animal Health, Animal Health Research Centre (CReSA), Autonomous University of Barcelona (UAB), Catalonia, Spain; 2 Institute of Agrifood Research and Technology (IRTA), Animal Health Program (CReSA), World Organization for Animal Health (WOAH) Collaborating Centre for the Research and Control of Emerging and Re-Emerging Swine Diseases in Europe, Autonomous University of Barcelona (UAB), Catalonia, Spain; 3 School of Agrifood and Forestry Science and Engineering (ETSEA), Department of Animal Production, University of Lleida, Catalonia, Spain; 4 Institute of Agrifood Research and Technology (IRTA), Animal Breeding and Genetics Program, Catalonia, Spain

**Keywords:** archeome, gut microbiota, metagenome-assembled genomes, metatranscriptomics, swine

## Abstract

**Introduction:**

The gut microbiota comprises a diverse and dynamic community of microorganisms that collectively enhance host metabolism, physiology, and overall functionality. In this context, the swine archaeome remains largely underexplored despite growing evidence that archaea may greatly influence host health. Advances in high-throughput approaches provide new opportunities to reveal the dynamics and composition of archaea. Herein, we uncover the taxonomic and functional landscape of the piglet archaeome during the weaning transition under multiple experimental conditions, integrating shotgun metagenomic and metatranscriptomic analyses to elucidate its contribution to gut microbial ecology.

**Methods:**

The seven experimental conditions included four antibiotic treatments for post-weaning diarrhoea (trimethoprim/sulfamethoxazole, colistin, gentamicin, amoxicillin), an oral vaccine, acidifiers in drinking water, and a no-intervention group. A total of 280 faecal samples were collected longitudinally one day before weaning (ST1), three days (ST2), two weeks (ST3), and four weeks (ST4) after the start of the treatment. Treatment was initiated eleven days after arrival at the experimental farm following the onset of clinical signs. Shotgun metagenomics was used to assess archaeal taxonomic diversity and recover archaeal metagenome-assembled genomes (aMAGs), while metatranscriptomics was integrated to assess differentially expressed genes at ST1, ST2, and ST4.

**Results:**

The results revealed archaea as the second most abundant microorganism, exhibiting a longitudinal increase in diversity over the experimental time. The most predominant genus was *Methanobrevibacter*, including *Methanobrevibacter smithii*. Eleven high-quality aMAGs were recovered, belonging to the Methanobacteriota and Thermoplasmatota phyla. Genome-inferred functional analyses revealed that the predominant metabolic processes included the biosynthesis of nucleic acids, amino acids, organic anions, and vitamins. Additional functional traits suggested potential roles in the degradation of sugars, amino acids, and antibiotics were also observed. Moreover, significant differences were detected on the archaeal metatranscriptome between the experimental groups treated with antibiotics and the rest of the groups, underscoring their response to changes in microbial interactions, substrate availability and, in some cases, direct effect of the antimicrobials on metabolic pathways.

**Discussion:**

Altogether, this study highlights the biological significance of archaeal dynamics during initial life stages and demonstrates how combining metagenomics and metatranscriptomics uncovers their functional potential and the pathways actively expressed in the piglets’ gut.

## 1 Introduction

The gastrointestinal tract is inhabited by a complex and dynamic microbial ecosystem that plays a central role in maintaining metabolic balance and intestinal health. This community contributes to essential processes including nutrient digestion and fermentation, vitamin synthesis, regulation of immune responses, preservation of intestinal structure and physiology, and protection against pathogenic microorganisms (Mach et al., 2015; Hu et al., 2024). This microbial diversity includes a wide range of biological entities, like bacteria, archaea, unicellular eukaryotes, and viruses. Among them, Archaea represent a distinct domain of prokaryotic microorganisms increasingly recognized as integral members of the human and animal microbiome, particularly present in the gastrointestinal tract and oral cavity (Ramayo-Caldas et al., 2020; Chen et al., 2024; Liu et al., 2025).

Archaea were first proposed as a separate lineage from bacteria in 1977, when Carl Woese and George Fox demonstrated through 16S rRNA comparisons that they were genetically more closely related to eukaryotes than to bacteria, despite their prokaryotic cell size and morphology (Woese and Fox, 1977). At that time, these microorganisms were considered methanogenic bacteria restricted to extreme environments with unique metabolic capabilities. In 1990, they were formally recognized as a distinct domain of life (Woese et al., 1990). Historically, archaeal diversity was described using broad superphyla such as Euryarchaeota, DPANN, and the TACK group (Thaumarchaeota, Aigarchaeota, Crenarchaeota, Korarchaeota) (Amils, 2011; Wang et al., 2021). However, recent genome-based frameworks from the Genome Taxonomy Database (GTDB) have revised this classification and proposed Methanobacteriota, Nanobdellota, and Thermoproteota phyla, respectively (Parks et al., 2026).

For decades, archaea remained underexplored due to their demanding culture requirements and the absence of known archaeal pathogens (Sun et al., 2020; Duller and Moissl-Eichinger, 2024). However, advances in molecular and high−throughput sequencing technologies have revealed that archaea are widespread across diverse environments, ranging from extreme habitats to soil, marine ecosystems, and host−associated microbiomes (Borrel et al., 2020; Wu et al., 2024). Although their ecological roles in the gut are not yet fully understood, emerging evidence suggests that specifically methanogenic archaea participate in the degradation of complex carbohydrates, detoxification of bacterial metabolites such as trimethylamine, methane production, and modulation of host immune responses (Yang et al., 2023; Duller et al., 2024). In humans, certain taxa have been associated with conditions such as periodontitis and intestinal methanogen overgrowth, particularly involving *Methanobrevibacter smithii* (Huynh et al., 2015). However, their potential pathogenic or beneficial role on human health is still under discussion.

In livestock, archaea have been specially studied in ruminants with a negative effect linked to feed efficiency and methane production mainly associated with *Mehanobrevibacter* spp (Xu et al., 2021). While bacterial communities have been extensively characterized in swine, the diversity, ecological significance, and functional contributions of the swine archaeome remains insufficiently understood (Deng et al., 2021; Chen et al., 2024). This knowledge gap is particularly relevant during the weaning transition, a critical period marked by dietary, environmental, and physiological changes, often associated with increased antimicrobial interventions (Li et al., 2018; Suriyaphol et al., 2021). Altogether, the impact of these factors on archaeal populations in piglets gut microbiome remains unclear.

Recent advances in omics methodologies have substantially improved the capacity to detect, classify, and functionally characterize archaeal populations within dynamic microbial ecosystems (Baker et al., 2020; Wu et al., 2024). Metagenomic sequencing enables the recovery of metagenome−assembled genomes (MAGs), facilitating the discovery of novel archaeal lineages, while metatranscriptomics provides insights into their active metabolic functions. To date, no studies have generated archaeal MAGs from piglets and integrated them with metatranscriptomic data to assess archaeal activity during early−life transition.

This study, conducted as part of a larger investigation (Guitart-Matas et al., 2024, 2025), aimed to characterize the taxonomic and functional diversity of the piglet archaeome under the effect of different treatments and conditions already implemented to reduce the risk of post-weaning diarrhoea (PWD). PWD is a complex, multifactorial disease causing significant economic losses in swine production worldwide. While viral and bacterial co-infections have been well documented (Garcias et al., 2024), the potential role of archaea in the disease pathogenesis has not been investigated. By leveraging a multi−omics approach, in which we identify the functional potential of archaeal communities through metagenomics, we also mapped metatranscriptomic reads from the same experimental setting to determine active and differentially expressed archaeal functions and metabolic pathways over time. This integrative framework provides new insights into the dynamics, ecological roles, and potential contributions of archaea within the piglet gut microbiota during the weaning transition.

## 2 Materials and methods

### 2.1 Experimental design

A total of 210 piglets from a farm in Catalonia (Spain) were selected for gut microbiome studies under different PWD treatments in October 2020. The farm of origin was appointed for previous records of diarrhoea outbreaks, and the experimental design was performed as previously described in Guitart-Matas et al., 2024. Briefly, 30 sows were randomly selected and after farrowing, 7 piglets per sow were ear-tagged to make a total of 210 piglets. Piglets were divided into seven groups, including one sibling per sow in each of the groups. One group remained at the farm of origin until the end of the experiment and received routine amoxicillin treatment (GG). The remaining six groups were transferred to an experimental farm from the Institute of Agrifood Research and Technology (IRTA) and exposed to different conditions: trimethoprim/sulfamethoxazole treatment (G1), colistin treatment (G2), commercial oral *Escherichia coli* vaccine (G3), gentamicin treatment (G4), control with phosphoric acid 75% in the drinking water (G5), and untreated group (G6).

Antibiotics were applied orally in water for five days when signs of diarrhoea appeared in individual animals, eleven days after the arrival at the experimental farm. Dosages and concentrations were determined by the summary of product characteristics (SmPC) of Methoxasol for the trimethoprim/sulfamethoxazole treatment (25 mg/kg/day, Genera Inc.), Apsasol for the colistin treatment (1.5×10^5^ international units (IU)/kg/day, Andrés Pintaluba, S.A.), and Gentavet for the gentamicin treatment (2×10^3^ IU/kg/day, Fatro S.p.A.). Vaccination with Coliprotec F4/F18 (Elanco GmbH) was applied orally in a single dose the day of arrival at the experimental farm. Faecal samples were retrieved aseptically directly from the animals (10 piglets per group) on four different sampling times and frozen instantly in dry ice: at the farm of origin the day before departure to the experimental farm and before treatment (ST1), three days post-treatment (ST2), two weeks (ST3), and four weeks post-treatment (ST4). Faecal samples were also collected from animals that remained at the farm of origin treated with amoxicillin at the same sampling times.

### 2.2 Ethical statement

Animals on the experimental farm were exposed to the same conditions as on the conventional farm and were allocated following legislation in animal welfare. Antimicrobial treatments followed the SmPC, and no disease was induced. The Ethics Committee for Animal Experimentation (CEEA) guidelines reviewed and authorized the procedures of this study with the ID number CEEA103/2018.

### 2.3 Shotgun metagenomics and metatranscriptomics sequencing

Total DNA from faecal samples was extracted using the QIAamp^®^ PowerFecal^®^ Pro DNA Kit (Qiagen, Hilden, Germany) according to the manufacturer’s instructions. Ten animals per group were selected for shotgun metagenomics sequencing, and nucleic acid was extracted for all sampling times (ST1, ST2, ST3, and ST4) and paired end sequenced (2 x 150 bp) on an Illumina NovaSeq 6000 platform at a 10 Gb sequencing depth (Novogene Bioinformatics Technology, Cambridge, United Kingdom).

Total RNA was extracted using the NucleoSpin™ RNA Stool (Macherey-Nagel™, Düren, Germany) from seven animals per experimental group at ST1, ST2, and ST4, following manufacturer’s instructions. When possible, these animals were selected to match the same individuals included in the metagenomic analyses. Total extracted RNA was also paired end sequenced (2 x 150 bp) for shotgun metatranscriptomics on an Illumina NovaSeq6000 platform at a 12 Gb sequencing depth (Centre Nacional d’Anàlisi Genòmic (CNAG-CRG), Catalonia, Spain). For detailed information about animals selected see BioProject PRJNA1010706.

As previously described (Guitart-Matas et al., 2024, 2025), shotgun metagenomics and metatranscriptomic reads were filtered with KneadData v0.7.7 software for quality control, using *Susscrofa*11.1 assembly for host decontamination (Langmead and Salzberg, 2012; Bolger et al., 2014). For metatranscriptomic reads, an additional step to remove ribosomal RNA (rRNA) was performed with SortMeRNA v4.3.6 with the Rfam and Silva databases (silva-bac-16s-id90, silva-arc-16s-id95, silva-bac-23s-id98, silva-arc-23s-id95, silva-euk-18s-id95, silva-euk-28s-id98), and the parameters: “*--out2 –paired_out –fastx*” (Griffiths-Jones, 2003; Kopylova et al., 2012). Kraken2 v2.1.2 software was chosen for archaeal taxonomic assignment against the maxikraken2_1903_140GB database at a 0.01 confidence score (Wood et al., 2019). The R microeco v1.5.0 package, based on the R6 class system, was used for data processing (Liu et al., 2021). Statistical differences in archaeal alpha diversity were evaluated using the Shannon, Chao1, and Observed indices, accounting for diversity richness and evenness (Shannon, 1948; Chao, 1984; Hu, 2011). The nonparametric Wilcoxon pairwise test was applied to compare Shannon alpha diversity indices between experimental groups and sampling times. The Benjamini-Hochberg (BH) method was applied to correct *P*-values for multiple comparisons (Benjamini and Hochberg, 1995). Beta diversity analyses were performed to assess differences in community composition across experimental groups and sampling times. Archaeal community dissimilarities were calculated using the Bray-Curtis distance metric from the normalized data by relative abundance, and ordination was conducted using Principal Coordinates Analysis (PCoA) (Bray and Curtis, 1957; Gower, 2015). Differences in beta diversity were evaluated with a permutational multivariate analysis of variance (PERMANOVA) using the *adonis2* function from the *vegan* v2.6–6 R package (999 permutations) between experimental groups and sampling times examining pairwise comparisons (Anderson, 2001; Oksanen et al., 2001). Relative abundance plots were done with *MicrobiomeStat* v1.4.1 and *ggplot2* v4.0.2 R packages (Wickham, 2016; Zhou et al., 2022).

### 2.4 Archaeal metagenome-assembled genomes

From paired reads, single sample assemblies were performed with MetaSPAdes assembler v3.15.5 (Nurk et al., 2017) and co-assemblies per experimental group and sampling time were generated with Megahit v1.0.2 (Li et al., 2015), as previously described (Guitart-Matas et al., 2025). Bins were generated and refined with the MetaWRAP software v1.3 from both single sample assemblies and co-assemblies setting a minimum completion of 70% (-c 70) and a maximum contamination of 10% (-x 10) (Uritskiy et al., 2018).

Final archaeal metagenomes were assessed with the “*lineage_wf*” workflow of CheckM v1.2.0 (Parks et al., 2015). Coverage was measured with CoverM v0.7.0 default relative abundance method (Aroney et al., 2025) and Prokka v1.15.6 was utilized for the calculation of tRNA counts and the identification of rRNA genes (Seemann, 2014). PhyloPhlAn v3.1.68 was used to generate a phylogenetic archaeal tree and the final maximum likelihood tree was constructed using RAxML v8.2.12 (Stamatakis, 2014; Asnicar et al., 2020). Functional annotation was performed with DRAM v1.5.0 with the following databases: Uniref90, PFAM-A, KOfam, and dbCAN-V10 (all downloaded in June 2023) and the R package *distillR* was used to transform raw archaeal annotations into multiple Genome Inferred Functional Traits (GIFTs) (Kanehisa et al., 2016; Alberdi and Langa, 2022).

### 2.5 Archaeal transcript abundances and differential expression analyses

For the quantification of archaeal transcript abundances, the cleaned metatranscriptomic reads were pseudoaligned with the ORFs encoded by the aMAGs using the Kallisto software v0.50.1 (Bray et al., 2016). The count abundance table was analysed for differential expression using the median of ratios for normalization with the *DESeq2* v1.52.0 Bioconductor package in R 4.5.2 (Love et al., 2014). Principal component analysis (PCA) allowed clustering and exclusion of two outliers from the trimethoprim/sulfamethoxazole- (G1, n=1) and gentamicin-treated (G4, n=1) groups at three days after treatment. Genes with fewer than 10 counts were removed prior to differential expression analysis, and genes with an adjusted *P*-value < 0.01 after multiple testing correction were considered differentially expressed. Differentially expressed genes (DEGs) were classified according to the sign of the log_2_-fold change estimate, with positive values considered up-regulated and negative values considered down-regulated. Similarity among groups was assessed using a signed Jaccard index based on direction-specific DEG sets, thus accounting for both gene overlap and direction of change. For DEGs with an assigned KEGG Orthology (KO) identifier derived from the aMAG functional annotation, functional classification was performed according to the corresponding KEGG pathway categories, and for each group and category, the number of DEGs and the mean log_2_-fold change were calculated (Kanehisa et al., 2016). DEG intersections across groups were visualized in R using the *ggplot2* v4.0.2 and *tidyverse* v2.0.0 packages in a custom UpSet-style plot, retaining only intersections with counts greater than 1 (Wickham, 2016; Wickham et al., 2019).

## 3 Results

### 3.1 Archaeal diversity and taxonomy analyses

**Figure 1:**
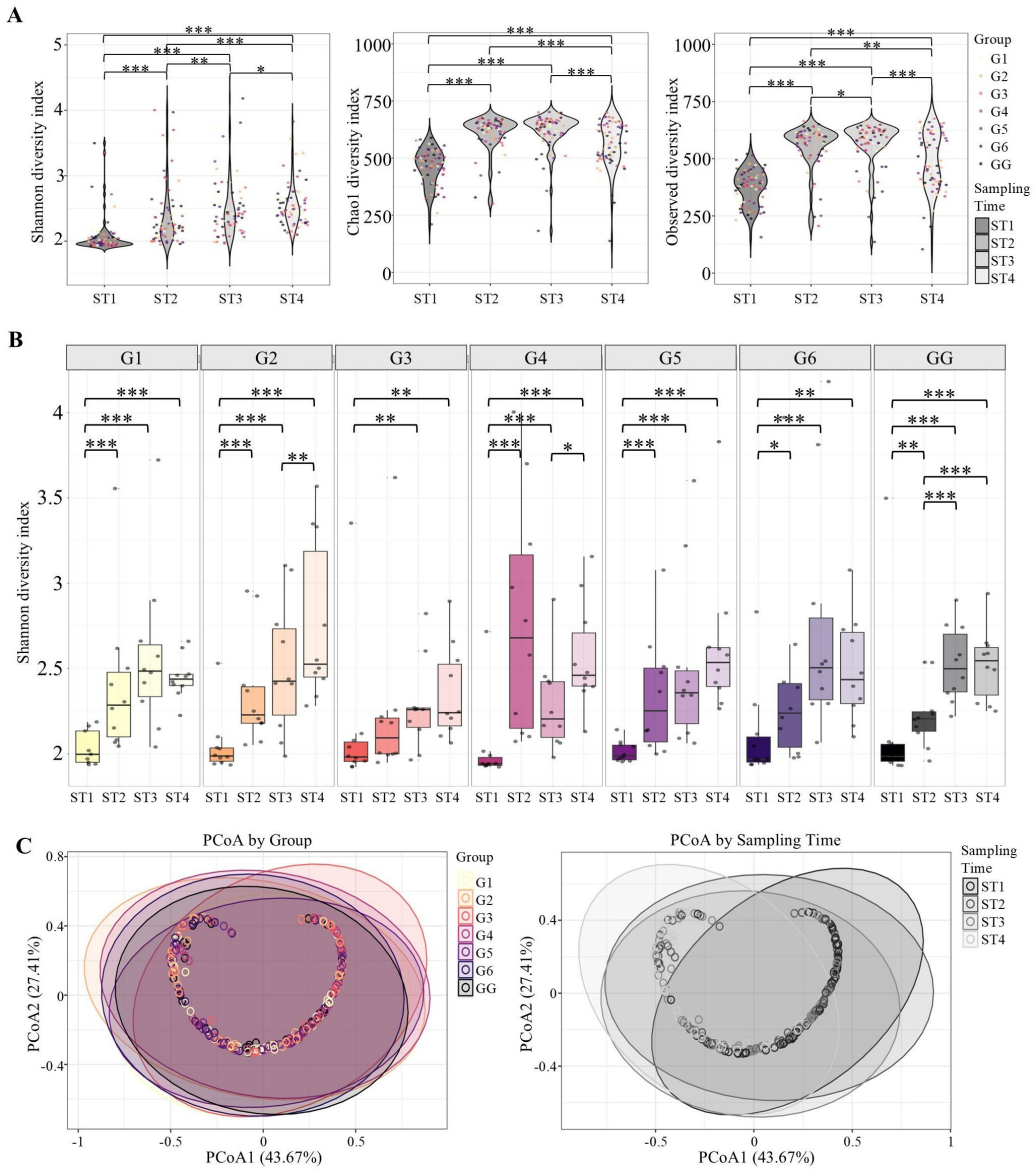
**(A)** Shannon, Chao1, and Observed diversity indices per sampling time of all experimental groups. **(B)** Shannon diversity represented by sampling time for each experimental group. **(C)** Principal Coordinates Analysis (PCoA) of archaeal community dissimilarities calculated with the beta diversity Bray-Curtis distance metric using the group and sampling time variables. Significant differences are highlighted at 0.01 (***), 0.05 (**), and 0.1 (*) significance levels. G1: trimethoprim/sulfamethoxazole, G2: colistin, G3: oral vaccination, G4: gentamicin, G5: untreated group with water acidification, G6: untreated group, GG: amoxicillin (farm of origin). ST1: one day before weaning, ST2: three days post-treatment, ST3: two weeks post-treatment, ST4: four weeks post-treatment.

Analyses of alpha diversity revealed significant differences across sampling times and experimental groups for the Shannon, Chao1, and Observed indices. Pairwise comparisons of all data between sampling times identified significant lower archaeal diversity one day before weaning (ST1) when compared to the diversity observed three days (ST2, *P* < 0.01), two weeks (ST3, *P* < 0.01), and four weeks (ST4, *P* < 0.01) post-treatment for all three diversity indices (**Figure 1A**). After weaning, a steady increase in archaeal diversity was also observed for all three alpha diversity indices. Shannon diversity index three days post-treatment (ST2) was significantly lower when compared to two weeks (ST3, *P* = 0.02) and four weeks (ST4, *P* < 0.01) post-treatment, while Chao1 and Observed indices showed lower archaeal diversity at three days (ST2, *P* < 0.01 and *P* = 0.011, respectively) and two weeks post-treatment (ST3, *P* < 0.01 for both indices) when compared to four weeks post-treatment (ST4) (**Figure 1A**).

In all experimental groups, archaeal diversity one day before weaning (ST1) was significantly lower when compared to sampling times after weaning (ST2, ST3, ST4, *P* < 0.1), except for the vaccinated group (G3) that showed significant lower archaeal diversity before weaning (ST1) when compared to two weeks (ST3, *P* = 0.044) and four weeks (ST4, *P* = 0.023) post-treatment, but not when comparing archaeal diversity three days after weaning (ST2, *P* = 0.158) (**Figure 1B**). Moreover, differences between sampling times after weaning were only observed in antibiotic-treated groups, except for the trimethoprim/sulfaphetoxazole-treated group (G1). The colistin-treated (G2) and gentamicin-treated (G4) groups showed significantly lower archaeal diversity two weeks after treatment (ST3) when compared to four weeks after treatment (ST4, *P* = 0.022 and *P* = 0.079, respectively), while the group treated with amoxicillin (GG) showed lower archaeal diversity three days after treatment (ST2) when compared to two weeks (ST3, *P* < 0.01) and four weeks (ST4, *P* < 0.01) post-treatment (**Figure 1B**). Pairwise comparisons between groups only identified a significant trend at two weeks post-treatment (ST3) with a lower archaeal diversity in the vaccinated group (G3) when compared with the untreated (G6, *P* = 0.069) and amoxicillin-treated (GG, *P* = 0.069) groups.

Beta diversity analyses did not reveal significant differences in microbial community composition across experimental groups (F = 1.09, R^2^ = 0.02, *P* = 0.316), indicating similar community profiles between groups. In contrast, an effect was observed on beta diversity when considering the distinct sampling times (F = 10.43, R^2^ = 0.10, *P* < 0.001). Pairwise comparisons revealed that stronger differences in community composition were observed between samples before weaning (ST1) and samples collected four weeks after treatment (ST4) (F = 26.33, R^2^ = 0.16, *P* = 0.001), while weakest differences were identified between samples collected three days after treatment (ST2) and two weeks after treatment (ST3) (F = 2.75, R^2^ = 0.02, *P* = 0.001). These contrasts between experimental groups and sampling times are represented in the PCoA ordination plot (**Figure 1C**).

**Figure 2:**
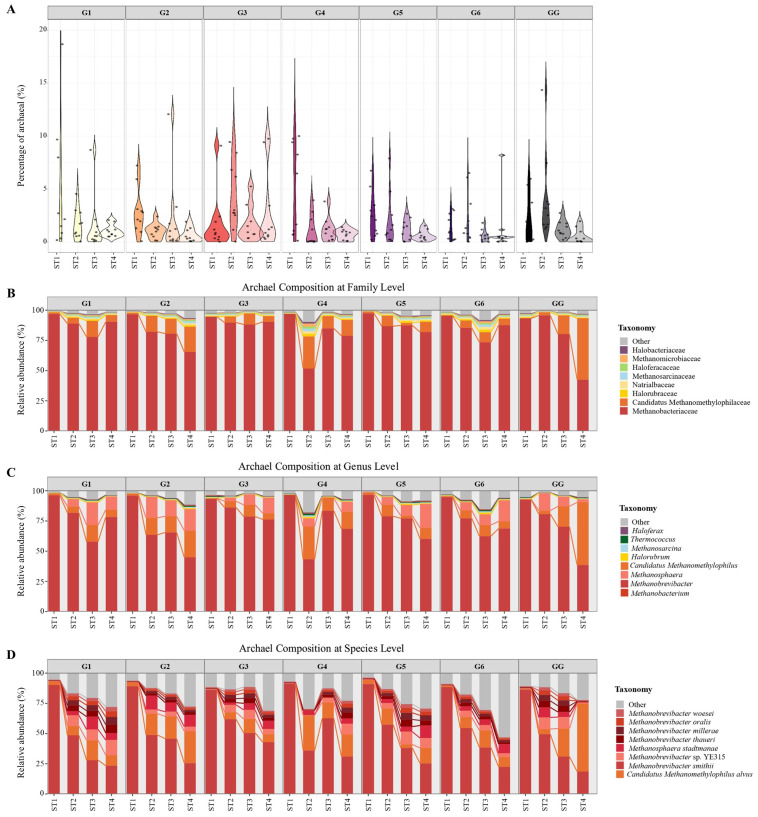
**(A)** Percentage of reads per experimental group and sampling time. Relative abundance of archaea in porcine microbiota at the **(B)** family, **(C)** genera, and **(D)** species level for the different experimental groups and sampling times: G1: trimethoprim/sulfamethoxazole, G2: colistin, G3: oral vaccination, G4: gentamicin, G5: untreated group with water acidification, G6: untreated group, GG: amoxicillin (farm of origin). ST1: one day before weaning, ST2: three days post-treatment, ST3: two weeks post-treatment, ST4: four weeks post-treatment. Data expressed in relative abundance (%) with no accounting for unclassified taxa.

Taxonomic classification with the Maxikraken database at a 1% confidence threshold accounted for an average of 1.97% (SD = 2.91%) of archaeal reads per sample (**Figure 2A**). Relative abundance patterns were studied at different taxonomic ranks (**Figures 2B–D**), corroborating the above observation of increasing archaeal diversity over sampling times. Before weaning (ST1), *Methanobacteriaceae* was the most represented family across all experimental groups, together with *Methanobrevibacter* genus and *M. smithii*. After weaning, larger differences in archaeal diversity were observed in the colistin- (G2), gentamicin- (G4), and amoxicillin-treated (GG) groups at the family and genus levels. These differences were especially pronounced three days after treatment (ST2) for the gentamicin-treated group (G4), with an increase of *Candidatus Methanomethylophilus* and *Methanosphaera* genera, including species such as *Candidatus Methanomethylophilus alvus*. Also, a higher relative abundance of the *Candidatus M. alvus* was observed in the colistin- (G2) and amoxicillin-treated (GG) groups four weeks after treatment (ST4), with multiple *Methanobrevibacter* species identified in all experimental groups (**Figure 2D**). However, *Methanobrevibacter* sp. AbM4 and *Methanobrevibacter boviskoreani* were more represented in the vaccinated group (G3), the untreated group (G6), and the trimethoprim/sulfamethoxazole- (G1) and amoxicillin-treated (GG) groups, but nearly unnoticeable in the other experimental groups. Also, the species *Methanobrevibacter woesei* and *Methanobrevibacter oralis* were detected in higher relative abundance in the trimethoprim/sulfamethoxazole- (G1) and gentamicin-treated (G4) groups and the group with water acidification (G5), compared to the other experimental groups (**Figure 2D**).

### 3.2 Archaeal metagenome-assembled genomes recovered

The assembly strategy performed here, combining bins generated from single assemblies and co-assemblies per experimental group and sampling time, followed by the refinement and de-replication modules, allowed the generation of 11 non-redundant aMAGs. Of these, 9 aMAGs (81.8%) were defined as high-quality MAGs according to the Minimum Information about a Metagenome-Assembled Genome (MIMAG) standards (> 90% completeness, < 5% contamination) (Bowers et al., 2017). Prior to de-replication, a cumulative total of 274 aMAGs were retrieved across the co-assemblies, representing an average of 1.56% (SD = 0.52%) of the total recovered bins per treatment group and sampling time.

The final collection with 11 aMAGs was characterized by a completeness mean of 92.12% (SD = 0.81%) and a contamination mean of 0.81% (SD = 1.00%). The mean size was 1.58 Mbp with a mean number of scaffolds of 166.27 (**Table 1**). Taxonomic classification identified 8 aMAGs as Methanobacteriota and 3 aMAGs as Thermoplasmatota. Besides, CoverM allowed the mapping of each individual sample to the aMAGs, and showed that the most prevalent aMAG across samples belonged to the *M. smithii* species (detected in n = 234, 83.57%), followed by *Methanomethylophilus alvus* (n = 151, 53.93%), *Methanosphaera stadtmanae* (n = 130, 46.43%), *Methanarcanum hacksteinii* (n = 122, 43.57%), and *Methanobrevibacter* sp900769095 (n = 121, 43.21%) (**Table 1**).

**Table 1:** Archaeal metagenome-assembled genomes (aMAGs) identified in this study.

aMAG ID	Completeness (%)	Contamination (%)	Genome size	Scaffolds	Species	Number of samples	Mean coverage	N50	Number of tRNAs	Presence 5S/16S/23S
*bin.484*	100.00	0.00	1804607	27	*Methanobrevibacter smithii*	234	0.96	176884	30	None
*bin.478*	100.00	0.00	1729050	83	*Methanobrevibacter* sp022775905	99	0.11	41349	26	16S
*bin.372*	99.73	0.00	1487216	73	*Methanobrevibacter* sp900769095	121	0.27	34221	25	16S
*bin.143*	97.33	0.00	1754273	139	*Methanosphaera stadtmanae*	130	0.09	19950	39	5S/16S/23S
*bin.662*	96.80	0.00	1605264	55	*Methanosphaera* sp900322125	24	0.21	45627	20	None
*bin.444*	96.63	1.76	1736537	153	*Methanobrevibacter boviskoreani*	16	0.42	15768	20	None
*bin.567*	95.92	0.00	1360316	43	*Methanarcanum hacksteinii*	122	0.04	53174	40	5S/16S
*bin.49*	90.46	2.08	1345830	209	*Methanomethylophilus alvus*	151	0.03	8474	33	5S
*bin.532*	90.01	2.48	1649258	458	SIG5 sp016297775	55	0.02	5175	38	5S
*bin.56*	73.47	1.60	1267850	224	*Methanobrevibacter* sp.	17	0.12	7072	16	5S/16S
*bin.86*	72.96	0.98	1588760	365	*Methanobrevibacter ruminantium*	14	0.05	5325	15	5S

For each aMAG, the table includes quality parameters, species-level taxonomic assignment, the number of samples in which it was detected, and its mean per-sample coverage.

**Figure 3:**
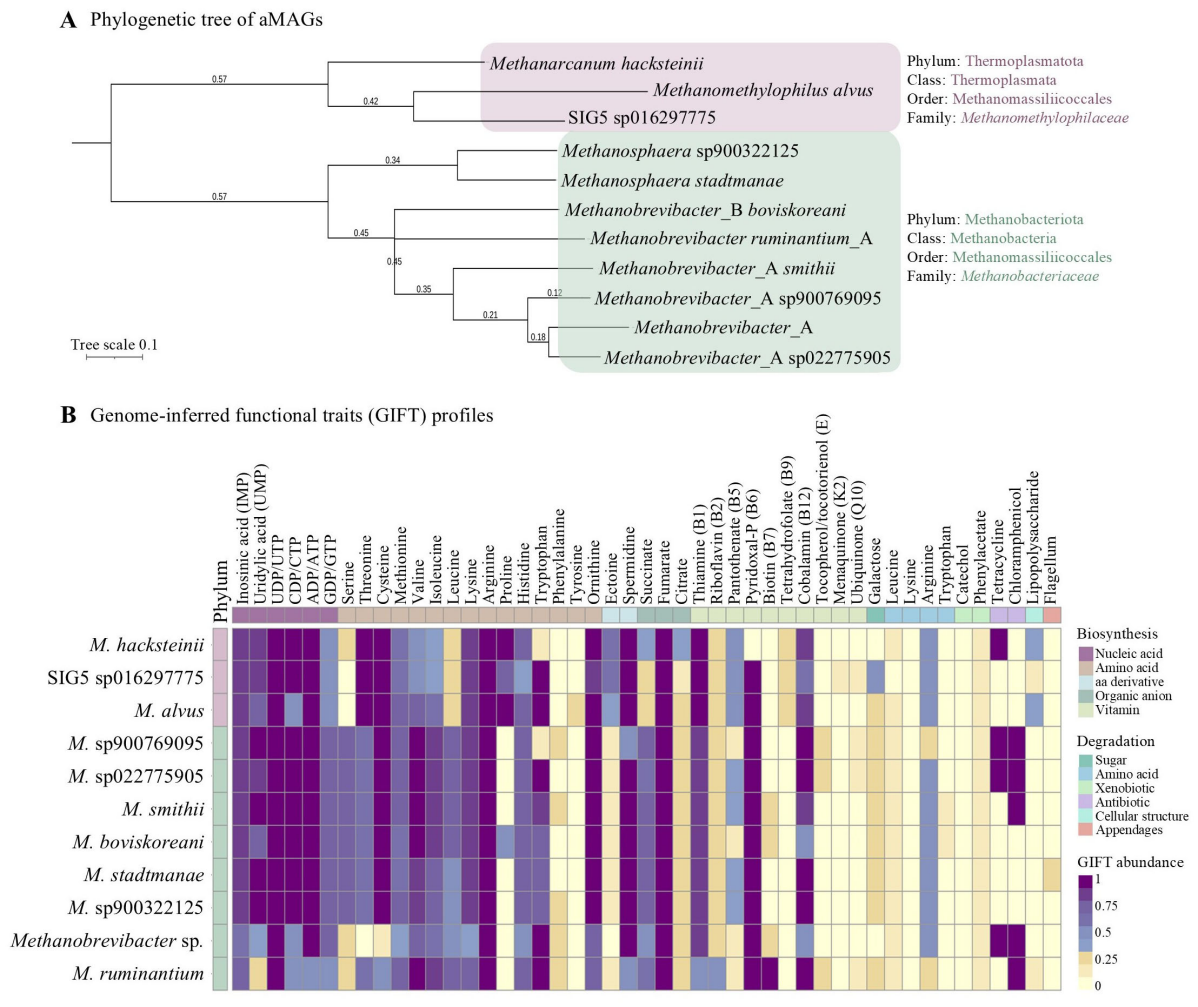
**(A)** Phylogenetic reconstruction of the collection of archaeal metagenome-assembled genomes (aMAGs). Different colours indicate different phyla. **(B)** Heatmap showing the abundance of the different genome-inferred functional traits (x-axis) represented in each aMAG (y-axis). Abundance for each genome-inferred functional trait (GIFT) is represented in a gradient scale.

The constructed phylogeny of the 11 aMAGs clustered different phyla together (**Figure 3A**). All aMAGs of the Thermoplasmatota phylum were classified within the Thermoplasmata class, Methanomassiliicoccales order, and *Methanomethylophilaceae* family. Similarly, aMAGs of the Methanobacteriota phylum belonged to the Methanobacteria class, Methanobacteriales order, and *Methanobacteriaceae* family (**Figure 3A**).

Inference of functional traits of the aMAGs allowed studying the metabolic potential of the archaeal lineages present in the piglets’ gut (**Figure 3B**). Most represented functions involved biosynthesis of nucleic acids, amino acids and derivatives, organic ions, and some vitamins. Some differences were observed between phyla, such as the higher number of traits involved in serine and leucine biosynthesis in most Methanobacteriota species compared to Thermoplasmatota species, and higher number of traits for proline and ectoine biosynthesis in Thermoplasmatota species (**Figure 3B**). Other relevant functional traits identified included the biosynthesis of succinate, fumarate, and citrate, and the vitamin biosynthesis of thiamine (B1), pantothenate (B5), pyridoxal-P (B6), and cobalamin (B12). Additionally, most prevalent functional traits related to metabolite degradation involved degradation of galactose sugar, arginine amino acid, and lipopolysaccharide, as well as pathways associated with two antibiotics, tetracycline and chloramphenicol. Further *in silico* analyses identified the specific KOs driving these antibiotic resistance mechanisms, including a predicted tetracycline resistance efflux pump (K18218) in four aMAGs and a chloramphenicol O-acetyltransferase type A (K19271) in five aMAGs (**Figure 3B**). Both of them co-occurred in the three *Methanobrevibacter* species that form a distinct cluster in the phylogenetic tree: *Methanobrevibacter* sp., *M.* sp900769095, and *M.* sp022775905 (**Figure 3**). To exclude potential contamination, a Protein Blast analysis of the nine protein sequences was conducted against the non-redundant protein sequence database (nr) comparing metrics against archaea (taxid:2157) and bacteria (taxid:2). The sequences exhibited high identity matches with protein sequences within the archaea taxid (mean sequence identity of 83.92% for K18218 and 74.82% for K19271 considering top 100 hits for each entry), while showing poor homology to bacterial sequences (mean sequence identity of 51.16% for K18218 and 34.27% for K19271) (Altschul, 1997).

### 3.3 Functional analyses

Metatranscriptomic data was mapped against the aMAGs database after trimming, host decontamination, and rRNA removal. A mean percentage of 5.97% reads (SD = 4.26%) pseudoaligned against the functional database. Principal component analysis (PCA) of the expression profiles clustered all samples, with the first two components explaining 66% of the total variance (44% by the first component and 22% by the second component).

**Figure 4:**
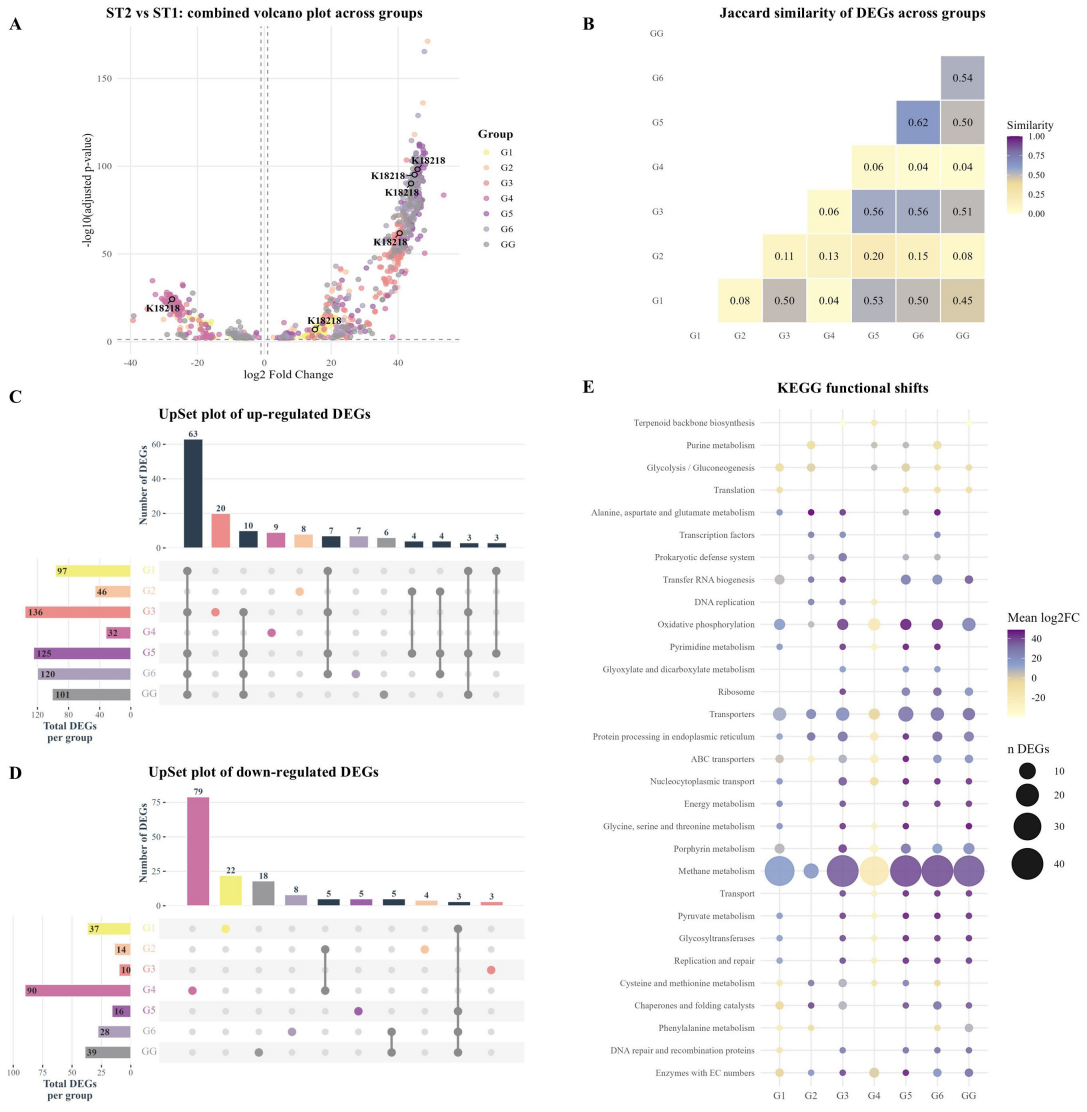
Differential expression and functional shifts in archaeal transcripts between three days post-treatment (ST2) and one day before weaning (ST1) across experimental groups. **(A)** Combined volcano plot showing all differentially expressed genes (DEGs) detected in each group, coloured by experimental group. **(B)** Pairwise Jaccard similarity among groups, summarizing the similarity in both DEG overlap and direction of change. **(C)** UpSet plot of up-regulated DEGs shared across groups. **(D)** UpSet plot of down-regulated DEGs shared across groups. Only intersections containing three or more genes are shown. **(E)** Bubble plot of KEGG functional shifts across groups; bubble size represents the number of DEGs assigned to each category and colour indicates the mean log_2_-fold change. G1: trimethoprim/sulfamethoxazole, G2: colistin, G3: oral vaccination, G4: gentamicin, G5: untreated group with water acidification, G6: untreated group, GG: amoxicillin (farm of origin).

Differential expression analyses were performed by comparing the DEGs of each experimental group between three days post-treatment (ST2) and the sampling time before weaning (ST1) to evaluate the short-term impact of the different experimental conditions after weaning. These comparisons revealed an early post-weaning shift in archaeal transcriptional activity across all experimental groups. Up-regulation of archaeal genes three days post-treatment (ST2) was most pronounced in the vaccinated group (G3, n = 136), the group with water acidification (G5, n = 125), the untreated group (G6, n = 120), followed by the amoxicillin- (GG, n = 101) and trimethoprim/sulfamethoxazole-treated (G1, n = 97) groups, whereas the colistin- (G2, n = 46) and gentamicin-treated (G4, n = 32) groups showed a more limited transcriptional shift. Besides, the gentamicin-treated group (G4) was the only group in which down-regulated genes (n = 90) outnumbered up-regulated genes, indicating a distinct early transcriptional response in this group (**Figure 4A**). Consistently, DEG profiles showed a higher overlap among the trimethoprim/sulfamethoxazole-treated group (G1), the vaccinated group (G3), the group with water acidification (G5), the untreated group (G6), and the amoxicillin-treated group (GG), while the colistin- (G2) and, especially, the gentamicin-treated (G4) groups were less similar to the remaining groups, supporting the presence of a common post-weaning response together with group-dependent deviations (**Figure 4B**).

This shared response was not universal, since the set of 63 common up-regulated genes was detected in five experimental groups (G1, G3, G5, G6, and GG), but not in the colistin- (G2) or gentamicin-treated (G4) groups (**Figure 4C**). In parallel, group-specific signatures were also evident, with the vaccinated group (G3) contributing the largest set of unique up-regulated genes, and the gentamicin-treated group (G4) the largest set of unique down-regulated genes (**Figures 4C, D**). Functional annotation showed that methane metabolism was the most consistently affected category and accounted for the highest number of DEGs, with positive shifts in all experimental groups except for the gentamicin-treated group (G4). Changes were also observed in oxidative phosphorylation, transporters, nucleocytoplasmic transport, and protein processing in endoplasmic reticulum, indicating that the weaning transition (ST2 vs ST1) involved broader changes in archaeal energy conservation and cellular transport functions (**Figure 4E**). Additionally, at this short-term period after weaning, the trimethoprim/sulfamethoxazole-treated group (G1) experienced negative shifts in chaperones and folding catalysts, DNA repair and recombination proteins, and a variety of enzymes, that were not detected in the other experimental groups (**Figure 4E**).

Because antimicrobial-associated annotations were detected in the aMAGs, we specifically evaluated whether K18218 and K19271 were represented in the archaeal metatranscriptomic dataset. Among these two annotations, only K18218 was detected in metatranscriptomic reads and passed the expression filters used for differential expression analysis. In the short-term comparison between three days post-treatment and before weaning (ST2 vs ST1), K18218 was up-regulated in all experimental groups except the colistin-treated group (G2), where it was not differentially expressed, and the gentamicin-treated group (G4), where it was down-regulated (**Figure 4A**). In the long-term comparison between four weeks post-treatment and before weaning (ST4 vs ST1), K18218 was also up-regulated in all groups except the colistin-treated group (G2) (**Supplementary Figure 1A**). When comparing four weeks post-treatment with three days post-treatment (ST4 vs ST2), K18218 was only significantly up-regulated in the gentamicin-treated group (G4), suggesting recovery from the early transcriptional suppression observed in this group.

This temporal pattern was reinforced when comparing DEGs between four weeks after treatment (ST4) and the sampling time before weaning (ST1), where the total number of DEGs increased in all experimental groups. This comparison showed transcriptional responses that were predominantly up-regulated across all experimental groups, including the gentamicin-treated group (G4), which no longer displayed the predominantly repressed profile observed three days after treatment (ST2) (**Supplementary Figures 1A, B**). However, still the trimethoprim/sulfamethoxazole-treated group (G1), the vaccinated group (G3), the group with water acidification (G5), the untreated group (G6), and the amoxicillin-treated group (GG) appeared to share more up-regulated genes (n = 51) between them, than when including colistin- (G2) and gentamicin-treated (G4) groups (**Supplementary Figure 1C**). Considering this longer-term impact comparison of the different conditions, the trimethoprim/sulfamethoxazole-treated group (G1) appeared with the highest number of unique down-regulated genes, showing negative shifts in chaperones and folding catalysts, multiple enzymes, ribosome and transfer RNA biogenesis, glycolysis, and different metabolisms, such as phenylalanine and purine (**Supplementary Figures 1D, E**). Overall, most prevalent archaeal up-regulated genes in all experimental groups at the end of the experiment (ST4) were still involved in methane metabolism, followed by oxidative phosphorylation and transporters, protein processing in endoplasmic reticulum, ribosome and chromosome-associated proteins, as well as general energy metabolism (**Supplementary Figure 1E**).

## 4 Discussion

The gut microbiome has been extensively described in most livestock species including pigs. While bacterial communities have been studied in detail and their contribution to host health has been widely identified, the functional roles of other microbial communities inhabiting the pig gut are still poorly understood. This is particularly true for Archaea, whose diversity, ecological relevance, and metabolic functionality remain unclear. While recent advances in omics technologies are helping to shed light on this underexplored domain of life, studies integrating metagenomic and metatranscriptomic data still remain scarce, with most existing work focusing primarily on predicting functional potential encoded in the archaeal genome repertoire. Herein, we have applied both approaches to unravel not only the archaeal community composition longitudinally, but also the functional changes along the weaning transition and the impact of different antimicrobial treatments in this domain.

In our experimental setting, the archaea composition increased in richness and diversity over the weaning period, probably driven by changes in diet and environment, and potentially influenced by the symbiotic interactions between archaeal and bacterial communities. However, this pattern differed from the rapid increase in bacterial community diversity observed right after weaning (ST2) that diminished to initial values four weeks after treatment (ST4) (Guitart-Matas et al., 2024). Similarly to other longitudinal studies, a drastic reduction in relative abundance of *M. smithii* was observed across all groups over time, independently of treatment, accompanied by an increase in other species such as *Candidatus Methanomethylophilus alvus* and other *Methanobrevibacter* spp (Federici et al., 2015; Gaio et al., 2022). In humans, *M. smithii*, an early coloniser of the gut, has been detected in colostrum and it is associated with the consumption of milk (Deng et al., 2022). Presumably, in our study as the diet shifted from milk to plant-based carbohydrates from one day before weaning (ST1) to four weeks after treatment (ST4), the ecological conditions favouring *M. smithii* may have become limiting, changing the fermentative metabolic requirements toward new metabolic networks, and favouring the replacement and enrichment of other methanogen species (Federici et al., 2015). In addition, the transition toward a more plant-based diet may increase the availability of methanol and other methylated compounds generated during bacterial fermentation, which could favour methylotrophic taxa such as *Methanosphaera* spp. and *Candidatus Methanomethylophilus* spp (Borrel et al., 2012; Volmer et al., 2023). Consistently with our results, two unique phyla, Methanobacteriota and Thermoplasmatota, have been reported in aMAGs from pig faecal samples in different European countries (Yang et al., 2024). However, this is the first study generating aMAGs belonging to species *M. hacksteinii, M. stadtmanae*, and *M. boviskoreani* from pigs in Europe. In other studies, one aMAG from swine gut is available for *M. alvi* in France (BioSample SAMEA110414552) and another for *M. ruminantuum* in Italy (BioSample SAMN43789130), while for *M. smithii* only two aMAGs are available from swine gut in Germany (BioSamples SAMEA110360391 and SAMEA110402134) and one in France (BioSample SAMEA110414419, accessed March 2026).

Altogether, the aMAG collection was dominated by methanogenic lineages, particularly *Methanobrevibacter* spp., *Candidatus Methanomethylophilus* spp., and *Methanosphaera* spp., indicating that the active archaeal fraction was largely composed of taxa whose metabolism depends on hydrogenotrophic or methylotrophic methanogenesis. Because methanogens consume hydrogen and other products of microbial fermentation through interspecies hydrogen transfer, the dietary and ecological restructuring associated with weaning is expected to alter not only methanogenesis itself, but also the broader network of energy conservation and metabolite exchange (Volmer et al., 2023). Therefore, the predominance of methane metabolism among the differentially expressed functions is biologically coherent with both the taxonomic and genomic results obtained. In this context, the coordinated changes observed in methane metabolism, oxidative phosphorylation, and transport-related functions likely reflect an active metabolic reorganization of the archaeal community in response to the post-weaning gut environment.

The archaeal transcriptome underwent a rapid functional rearrangement after weaning, mainly characterized by the activation of methanogenesis-associated and other energy-related functions, in line with previous studies showing that weaning reshapes archaeal diversity, community structure, and functional potential in pigs, and that archaeal succession accompanies the broader ecological transition of the gut microbiota during this period (Federici et al., 2015; Deng et al., 2022; Xiong et al., 2022).

Several studies have tried to determine the effect of different antimicrobials on the archaeal populations. Very few studies have performed *in vitro* experiments to assess the minimal inhibitory concentration (MIC) of different families of antimicrobials against culturable archaea, especially methanogenic species (Khelaifia and Drancourt, 2012). These experiments have provided valuable insights into their susceptibility to certain antimicrobials, including metronidazole and fusidic acid. However, most of the reports are based on indirect or empirical observations, such as metagenomic shifts following antimicrobial exposure or changes in fermentation parameters in complex ecosystems (Moissl-Eichinger et al., 2018). In our dataset, the effect of treatment appeared secondary to the effect of time after weaning, since beta diversity did not differ significantly across experimental groups but changed significantly across sampling times. Nevertheless, differences between post-weaning sampling times were detected only in antibiotic-treated groups, indicating that antimicrobial exposure may have modulated the archaeal trajectory during this transition in a treatment-dependent manner.

The between-group differences observed shortly after treatment suggest that this general post-weaning response was modulated by these specific interventions. The clearest example was the gentamicin-treated group (G4), which showed the most divergent early transcriptional profile, with predominant down-regulation and loss of the shared induced signature detected in the trimethoprim/sulfamethoxazole-treated group (G1), the vaccinated group (G3), the group with water acidification (G5), the untreated group (G6), and the amoxicillin-treated group (GG). Although gentamicin primarily targets bacterial ribosomes and is not expected to directly inhibit most gut archaea, this result is biologically plausible in the context of the gut ecosystem. Methanogenic archaea depend on bacterial fermentation products, including hydrogen, formate, methanol and other methylated compounds; therefore, perturbation of bacterial partners may indirectly alter archaeal energy metabolism and methanogenesis-related transcription (Volmer et al., 2023). This interpretation is consistent with the broader bacterial microbiome disruption previously observed in the gentamicin-treated group in the companion analysis of this experimental cohort (Guitart-Matas et al., 2024). Interestingly, studies have shown that methanogenic archaea exhibit resistance to many antibiotics, especially those targeting the cell wall or bacterial ribosomes, and *M. smithii* has been reported to be resistant to gentamicin *in vitro* (Dridi et al., 2011; Khelaifia and Drancourt, 2012). This result is particularly relevant because the gentamicin-treated group (G4) had also shown the most evident taxonomic deviation three days post-treatment (ST2), with an increase in *Candidatus Methanomethylophilus* spp. and *Methanosphaera* spp., indicating that both community composition and transcriptional activity departed from the general post-weaning pattern in this group. However, the later predominance of up-regulated genes in the gentamicin-treated group (G4), together with the overall increase in DEGs across groups over time, suggests that the repressed pattern detected three days after treatment (ST2) was transient rather than sustained. This temporal trajectory is compatible with a short-term treatment-associated perturbation, potentially mediated indirectly through changes in bacterial partners, substrate availability, and hydrogen or methylated-compound fluxes, rather than with a stable direct suppression of archaeal activity by gentamicin. This interpretation is also in agreement with previous evidence showing that methanogenic archaea are often broadly less susceptible than bacteria to conventional antibiotics and with piglet studies reporting limited archaeome changes after antimicrobial exposure (Dridi et al., 2011; Khelaifia and Drancourt, 2012; Zeineldin et al., 2021). Notably, this treatment-specific modulation was not restricted to the gentamicin-treated group (G4), since the colistin-treated group (G2) also showed a more limited early transcriptional response and lacked the shared induced signature detected in the trimethoprim/sulfamethoxazole-treated group (G1), the vaccinated group (G3), the group with water acidification (G5), the untreated group (G6), and the amoxicillin-treated group (GG). Together with the diversity shifts observed across post-weaning sampling times in the colistin- (G2), gentamicin- (G4), and amoxicillin-treated (GG) groups, these results suggest that antimicrobial exposure did not impose a common archaeal response, but rather transiently altered the post-weaning archaeal trajectory in a treatment-dependent manner. Interpretation of the amoxicillin-treated group (GG) should also be made with caution, since this group remained at the farm of origin and therefore antibiotic exposure cannot be fully disentangled from environmental and management differences relative to the groups transferred to the experimental facility.

Similarly, the trimethoprim/sulfamethoxazole-treated group (G1) experimented down-regulation of chaperone and folding catalyst functions, consistent with its mechanism of inhibiting folic acid synthesis, which unlike in the gentamicin-treated group (G4), it was sustained over time. Folate biosynthesis disruption is known in bacteria to impair amino acid production and to down-regulate protein folding pathways, including chaperone dependent systems. For instance, folate stress caused by subinhibitory concentrations of trimethoprim/sulfamethoxazole in *Acinetobacter baumannii* has demonstrated to suppress the chaperone-usher assembly system showing a direct link between folate depletion and reduced protein folding capacity (Moon et al., 2017). It is reasonable to expect a similar response in archaeal communities given that some archaeal protein folding systems are represented by chaperonins which share many conserved structural and functional motifs with bacterial chaperonins (Techtmann and Robb, 2010). In addition, sulphonamides are known to inhibit archaeal beta and gamma class carbonic anhydrases disrupting microbial metabolism, highlighting that archaeal enzymes can be directly targeted by sulphonamides (Zimmerman et al., 2004).

Finally, recent studies have highlighted the archaeome as a promising reservoir for antibiotic discovery. Large scale proteomic and genomic screening has revealed that the archaeal community encodes diverse antimicrobial molecules including peptidoglycan degrading proteins and encrypted antimicrobial peptides, termed archaeasin (Torres et al., 2025). In addition, recent experimental evidence has demonstrated the presence of functional multidrug efflux pumps in archaea, expanding the known repertoire of archaeal mechanisms potentially involved in antimicrobial tolerance (Fakhoury et al., 2024). Within this context, our functional analyses inferred the presence of some interesting antimicrobial-associated annotations, including chloramphenicol O-acetyltransferase type A (K19271) and tetracycline resistance efflux pump (K18218), two antibiotics considered chemically stable (Zhang et al., 2022; Semenova et al., 2025). The detection of these annotations suggests that archaeal communities may harbour uncharacterised enzymatic systems potentially involved in antimicrobial tolerance or detoxification, although their biological activity cannot be inferred from genomic annotation alone. This is particularly relevant for K18218, which was the only antimicrobial-associated annotation detected in the metatranscriptomic dataset. Since neither tetracycline nor chloramphenicol were administered in this experiment, K18218 expression should not be interpreted as a compound-specific response to tetracycline. Indeed, tetracycline-associated efflux proteins and multidrug transporters may also contribute to broader physiological processes, including ion homeostasis, stress responses, detoxification of endogenous metabolites, membrane-associated functions and ecological fitness (Krulwich et al., 2001; Martinez et al., 2009; Hong et al., 2014). Therefore, the transcriptional detection of K18218 in our dataset may reflect a broader archaeal stress or homeostatic response during the post-weaning transition rather than a specific response to tetracycline exposure. Such findings open the door to further explore these novel metabolic pathways for future applications in bioremediation and antimicrobial resistance mitigation.

Taken together, this study describes longitudinally the archaeal community dynamics during the weaning transition in piglets, revealing that this domain undergoes marked taxonomic and functional restructuring during early life. The archaeal ecosystem was characterized by an increase in richness and diversity over time, a progressive replacement of *M. smithii* by other methanogenic taxa, and a predominance of methanogenesis-related functions that were transcriptionally reshaped after weaning. Although the effect of antimicrobial treatments was less pronounced than the effect of time, specific interventions transiently altered the archaeal trajectory in a treatment-dependent manner, highlighting that archaeal communities may respond to both ecological perturbations associated with microbial interactions and substrate fluxes, including, in some cases, potential direct effects on archaeal metabolic functions. Overall, our findings position archaea as active and dynamic members of the pig gut microbiome, contributing to its metabolic adaptation during a critical developmental stage. By combining metagenomics and metatranscriptomics, this work expands current knowledge of the swine archaeome and provides the basis for future studies aimed at disentangling the ecological role, functional relevance, and applied potential of gut archaea in animal health, resilience, and microbiome-targeted interventions.

## Data Availability

The datasets presented in this study can be found in online repositories. The names of the repository/repositories and accession number(s) can be found below: https://www.ncbi.nlm.nih.gov/bioproject/?term=1010706.

## Supplementary material

Supplementary Figure 1 Differential expression and functional shifts in archaeal transcripts between four weeks post-treatment (ST2) and one day before weaning (ST1) across experimental groups. **(A)** Combined volcano plot showing all differentially expressed genes (DEGs) detected in each group, coloured by experimental group. **(B)** Pairwise Jaccard similarity among groups, summarizing the similarity in both DEG overlap and direction of change. **(C)** UpSet plot of up-regulated DEGs shared across groups. **(D)** UpSet plot of down-regulated DEGs shared across groups. Only intersections containing three or more genes are shown. **(E)** Bubble plot of KEGG functional shifts across groups; bubble size represents the number of DEGs assigned to each category and colour indicates the mean log_2_-fold change. G1: trimethoprim/sulfamethoxazole, G2: colistin, G3: oral vaccination, G4: gentamicin, G5: untreated group with water acidification, G6: untreated group, GG: amoxicillin (farm of origin).

## References

[B1] Alberdi A. Langa J. (2022). distillR: R package for distilling functional annotations of bacterial genomes and metagenomes.

[B2] Altschul S. (1997). Gapped BLAST and PSI-BLAST: a new generation of protein database search programs. Nucleic Acids Res. 25, 3389–3402. doi: 10.1093/nar/25.17.3389 9254694 PMC146917

[B3] Amils R. (2011). “ Euryarchaeota,” in Encyclopedia of Astrobiology ( Springer Berlin Heidelberg, Berlin, Heidelberg), 515–515. doi: 10.1007/978-3-642-11274-4_541

[B4] Anderson M. J. (2001). A new method for non‐parametric multivariate analysis of variance. Austral Ecol. 26, 32–46. doi: 10.1111/j.1442-9993.2001.01070.pp.x 40046247

[B5] Aroney S. T. N. Newell R. J. P. Nissen J. N. Camargo A. P. Tyson G. W. Woodcroft B. J. (2025). CoverM: read alignment statistics for metagenomics. Bioinformatics 41 (4), btaf147. doi: 10.1093/bioinformatics/btaf147 40193404 PMC11993303

[B6] Asnicar F. Thomas A. M. Beghini F. Mengoni C. Manara S. Manghi P. . (2020). Precise phylogenetic analysis of microbial isolates and genomes from metagenomes using PhyloPhlAn 3.0. Nat. Commun. 11, 2500. doi: 10.1038/s41467-020-16366-7 32427907 PMC7237447

[B7] Baker B. J. De Anda V. Seitz K. W. Dombrowski N. Santoro A. E. Lloyd K. G. (2020). Diversity, ecology and evolution of Archaea. Nat. Microbiol. 5, 887–900. doi: 10.1038/s41564-020-0715-z 32367054

[B8] Benjamini Y. Hochberg Y. (1995). Controlling the false discovery rate: a practical and powerful approach to multiple testing. J. R. Stat. Soc Ser. B. Stat. Methodol. 57, 289–300. doi: 10.1111/j.2517-6161.1995.tb02031.x 40046247

[B9] Bolger A. M. Lohse M. Usadel B. (2014). Trimmomatic: a flexible trimmer for Illumina sequence data. Bioinformatics 30, 2114–2120. doi: 10.1093/bioinformatics/btu170 24695404 PMC4103590

[B10] Borrel G. Brugère J.-F. Gribaldo S. Schmitz R. A. Moissl-Eichinger C. (2020). The host-associated archaeome. Nat. Rev. Microbiol. 18, 622–636. doi: 10.1038/s41579-020-0407-y 32690877

[B11] Borrel G. Harris H. M. B. Tottey W. Mihajlovski A. Parisot N. Peyretaillade E. . (2012). Genome sequence of “*Candidatus Methanomethylophilus alvus*” Mx1201, a methanogenic archaeon from the human gut belonging to a seventh order of methanogens. J. Bacteriol. 194, 6944–6945. doi: 10.1128/JB.01867-12 23209209 PMC3510639

[B12] Bowers R. M. Kyrpides N. C. Stepanauskas R. Harmon-Smith M. Doud D. Reddy T. B. K. . (2017). Minimum information about a single amplified genome (MISAG) and a metagenome-assembled genome (MIMAG) of bacteria and archaea. Nat. Biotechnol. 35, 725–731. doi: 10.1038/nbt.3893 28787424 PMC6436528

[B13] Bray J. R. Curtis J. T. (1957). An ordination of the upland forest communities of southern Wisconsin. Ecol. Monogr. 27, 325–349. doi: 10.2307/1942268 34212107 PMC8221127

[B14] Bray N. L. Pimentel H. Melsted P. Pachter L. (2016). Near-optimal probabilistic RNA-seq quantification. Nat. Biotechnol. 34, 525–527. doi: 10.1038/nbt.3519 27043002

[B15] Chao A. Chiu C. (2016). Nonparametric estimation and comparison of species richness. In: Encyclopedia of Life Sciences. John Wiley & Sons, Ltd., pp. 1–11. doi: 10.1002/9780470015902.a0026329

[B16] Chen Q. Lyu W. Pan C. Ma L. Sun Y. Yang H. . (2024). Tracking investigation of archaeal composition and methanogenesis function from parental to offspring pigs. Sci. Total Environ. 927, 172078. doi: 10.1016/j.scitotenv.2024.172078 38582109

[B17] Deng F. Li Y. Peng Y. Wei X. Wang X. Howe S. . (2021). The diversity, composition, and metabolic pathways of archaea in pigs. Animals 11, 2139. doi: 10.3390/ani11072139 34359268 PMC8300674

[B18] Deng F. Peng Y. Zhang Z. Howe S. Wu Z. Dou J. . (2022). Weaning time affects the archaeal community structure and functional potential in pigs. Front. Microbiol. 13. doi: 10.3389/fmicb.2022.845621 35387077 PMC8979004

[B19] Dridi B. Fardeau M.-L. Ollivier B. Raoult D. Drancourt M. (2011). The antimicrobial resistance pattern of cultured human methanogens reflects the unique phylogenetic position of archaea. J. Antimicrob. Chemother. 66, 2038–2044. doi: 10.1093/jac/dkr251 21680581

[B20] Duller S. Moissl-Eichinger C. (2024). Archaea in the human microbiome and potential effects on human infectious disease. Emerg. Infect. Dis. 30 (8), 1505–1513. doi: 10.3201/eid3008.240181 39043386 PMC11286065

[B21] Duller S. Vrbancic S. Szydłowski Ł. Mahnert A. Blohs M. Predl M. . (2024). Targeted isolation of *Methanobrevibacter* strains from fecal samples expands the cultivated human archaeome. Nat. Commun. 15, 7593. doi: 10.1038/s41467-024-52037-7 39217206 PMC11366006

[B22] Fakhoury A. A. Thompson T. P. Rahman K. M. Megaw J. McAteer M. I. Skvortsov T. . (2024). Identification and characterisation of two functional antibiotic MATE efflux pumps in the archaeon *Halorubrum amylolyticum*. NPJ Antimicrobials Resistance 2, 21. doi: 10.1038/s44259-024-00036-5 39843964 PMC11721430

[B23] Federici S. Miragoli F. Pisacane V. Rebecchi A. Morelli L. Callegari M. L. (2015). Archaeal microbiota population in piglet feces shifts in response to weaning: *Methanobrevibacter smithii* is replaced with *Methanobrevibacter boviskoreani*. FEMS Microbiol. Lett. 362 (10), fnv064. doi: 10.1093/femsle/fnv064 25903267

[B24] Gaio D. DeMaere M. Z. Anantanawat K. Eamens G. J. Falconer L. Chapman T. A. . (2022). Phylogenetic diversity analysis of shotgun metagenomic reads describes gut microbiome development and treatment effects in the post-weaned pig. PloS One 17, e0270372. doi: 10.1371/journal.pone.0270372 35749534 PMC9232140

[B25] Garcias B. Migura-Garcia L. Giler N. Martín M. Darwich L. (2024). Differences in enteric pathogens and intestinal microbiota between diarrheic weaned piglets and healthy penmates. Vet. Microbiol. 295, 110162. doi: 10.1016/j.vetmic.2024.110162 38941767

[B26] Gower J. C. (2015). “ Principal coordinates analysis,” in Wiley Statsref: Statistics Reference Online. (Hoboken, New Jersey: Wiley), 1–7. doi: 10.1002/9781118445112.stat05670.pub2

[B27] Griffiths-Jones S. (2003). Rfam: an RNA family database. Nucleic Acids Res. 31, 439–441. doi: 10.1093/nar/gkg006 12520045 PMC165453

[B28] Guitart-Matas J. Ballester M. Fraile L. Darwich L. Giler-Baquerizo N. Tarres J. . (2024). Gut microbiome and resistome characterization of pigs treated with commonly used post-weaning diarrhea treatments. Anim. Microbiome 6, 24. doi: 10.1186/s42523-024-00307-6 38702766 PMC11067243

[B29] Guitart-Matas J. Vera-Ponce de León A. Pope P. B. Hvidsten T. R. Fraile L. Ballester M. . (2025). Multi-omics surveillance of antimicrobial resistance in the pig gut microbiome. Anim. Microbiome 7, 65. doi: 10.1186/s42523-025-00418-8 40528272 PMC12172226

[B30] Hong H. Jung J. Park W. (2014). Plasmid-encoded tetracycline efflux pump protein alters bacterial stress responses and ecological fitness of *Acinetobacter oleivorans*. PloS One 9, e107716. doi: 10.1371/journal.pone.0107716 25229538 PMC4167995

[B31] Hu J. Chen J. Ma L. Hou Q. Zhang Y. Kong X. . (2024). Characterizing core microbiota and regulatory functions of the pig gut microbiome. ISME J. 18 (1), wrad037. doi: 10.1093/ismejo/wrad037 38366194 PMC10873858

[B32] Hu L. (2011). How to appropriately choose observed indexes. J. Chin. Integr. Med. 9, 491–494. doi: 10.3736/jcim20110505 21565134

[B33] Huynh H. T. T. Pignoly M. Nkamga V. D. Drancourt M. Aboudharam G. (2015). The repertoire of archaea cultivated from severe periodontitis. PloS One 10, e0121565. doi: 10.1371/journal.pone.0121565 25830311 PMC4382158

[B34] Kanehisa M. Sato Y. Kawashima M. Furumichi M. Tanabe M. (2016). KEGG as a reference resource for gene and protein annotation. Nucleic Acids Res. 44, D457–D462. doi: 10.1093/nar/gkv1070 26476454 PMC4702792

[B35] Khelaifia S. Drancourt M. (2012). Susceptibility of archaea to antimicrobial agents: applications to clinical microbiology. Clin. Microbiol. Infection 18, 841–848. doi: 10.1111/j.1469-0691.2012.03913.x 22748132

[B36] Kopylova E. Noé L. Touzet H. (2012). SortMeRNA: fast and accurate filtering of ribosomal RNAs in metatranscriptomic data. Bioinformatics 28, 3211–3217. doi: 10.1093/bioinformatics/bts611 23071270

[B37] Krulwich T. A. Jin J. Guffanti A. A. Bechhofer H. (2001). Functions of tetracycline efflux proteins that do not involve tetracycline. J. Mol. Microbiol. Biotechnol. 3, 237–246. 11321579

[B38] Langmead B. Salzberg S. L. (2012). Fast gapped-read alignment with Bowtie 2. Nat. Methods 9, 357–359. doi: 10.1038/nmeth.1923 22388286 PMC3322381

[B39] Li Y. Guo Y. Wen Z. Jiang X. Ma X. Han X. (2018). Weaning stress perturbs gut microbiome and its metabolic profile in piglets. Sci. Rep. 8, 18068. doi: 10.1038/s41598-018-33649-8 30584255 PMC6305375

[B40] Li D. Liu C.-M. Luo R. Sadakane K. Lam T.-W. (2015). MEGAHIT: an ultra-fast single-node solution for large and complex metagenomics assembly via succinct de Bruijn graph. Bioinformatics 31, 1674–1676. doi: 10.1093/bioinformatics/btv033 25609793

[B41] Liu C. Cui Y. Li X. Yao M. (2021). microeco: an R package for data mining in microbial community ecology. FEMS Microbiol. Ecol. 97 (2), fiaa255. doi: 10.1093/femsec/fiaa255 33332530

[B42] Liu H.-Y. Li S. Ogamune K. J. Ahmed A. A. Kim I. H. Zhang Y. . (2025). Fungi in the gut microbiota: interactions, homeostasis, and host physiology. Microorganisms 13, 70. doi: 10.3390/microorganisms13010070 39858841 PMC11767893

[B43] Love M. I. Huber W. Anders S. (2014). Moderated estimation of fold change and dispersion for RNA-seq data with DESeq2. Genome Biol. 15, 550. doi: 10.1186/s13059-014-0550-8 25516281 PMC4302049

[B44] Mach N. Berri M. Estellé J. Levenez F. Lemonnier G. Denis C. . (2015). Early‐life establishment of the swine gut microbiome and impact on host phenotypes. Environ. Microbiol. Rep. 7, 554–569. doi: 10.1111/1758-2229.12285 25727666

[B45] Martinez J. L. Sánchez M. B. Martínez-Solano L. Hernandez A. Garmendia L. Fajardo A. . (2009). Functional role of bacterial multidrug efflux pumps in microbial natural ecosystems. FEMS Microbiol. Rev. 33, 430–449. doi: 10.1111/j.1574-6976.2008.00157.x 19207745

[B46] Moissl-Eichinger C. Pausan M. Taffner J. Berg G. Bang C. Schmitz R. A. (2018). Archaea are interactive components of complex microbiomes. Trends Microbiol. 26, 70–85. doi: 10.1016/j.tim.2017.07.004 28826642

[B47] Moon K. H. Weber B. S. Feldman M. F. (2017). Subinhibitory concentrations of trimethoprim and sulfamethoxazole prevent biofilm formation by *Acinetobacter baumannii* through inhibition of Csu pilus expression. Antimicrob. Agents Chemother. 61 (9). doi: 10.1128/AAC.00778-17 28674047 PMC5571315

[B48] Nurk S. Meleshko D. Korobeynikov A. Pevzner P. A. (2017). metaSPAdes: a new versatile metagenomic assembler. Genome Res. 27, 824–834. doi: 10.1101/gr.213959.116 28298430 PMC5411777

[B49] Oksanen J. Simpson G. L. Blanchet F. G. Kindt R. Legendre P. Minchin P. R. . (2025). vegan: community ecology package. CRAN: Contributed Packages. doi: 10.32614/CRAN.package.vegan

[B50] Parks D. H. Chaumeil P.-A. Mussig A. J. Rinke C. ChuvoChina M. Hugenholtz P. (2026). GTDB release 10: a complete and systematic taxonomy for 715 230 bacterial and 17 245 archaeal genomes. Nucleic Acids Res. 54, D743–D754. doi: 10.1093/nar/gkaf1040 41123020 PMC12807784

[B51] Parks D. H. Imelfort M. Skennerton C. T. Hugenholtz P. Tyson G. W. (2015). CheckM: assessing the quality of microbial genomes recovered from isolates, single cells, and metagenomes. Genome Res. 25, 1043–1055. doi: 10.1101/gr.186072.114 25977477 PMC4484387

[B52] Ramayo-Caldas Y. Prenafeta-Boldú F. Zingaretti L. M. Gonzalez-Rodriguez O. Dalmau A. Quintanilla R. . (2020). Gut eukaryotic communities in pigs: diversity, composition and host genetics contribution. Anim. Microbiome 2, 18. doi: 10.1186/s42523-020-00038-4 33499953 PMC7807704

[B53] Seemann T. (2014). Prokka: rapid prokaryotic genome annotation. Bioinformatics 30, 2068–2069. doi: 10.1093/bioinformatics/btu153 24642063

[B54] Semenova Y. Makalkina L. Glushkova N. Gaipov A. (2025). Tetracyclines in the modern era: Global consumption, antimicrobial resistance, environmental occurrence, and degradation techniques. Antibiotics 14, 1183. doi: 10.3390/antibiotics14121183 41463684 PMC12729376

[B55] Shannon C. E. (1948). A mathematical theory of communication. Bell System Tech. J. 27, 379–423. doi: 10.1002/j.1538-7305.1948.tb01338.x 41531421

[B56] Stamatakis A. (2014). RAxML version 8: a tool for phylogenetic analysis and post-analysis of large phylogenies. Bioinformatics 30, 1312–1313. doi: 10.1093/bioinformatics/btu033 24451623 PMC3998144

[B57] Sun Y. Liu Y. Pan J. Wang F. Li M. (2020). Perspectives on cultivation strategies of archaea. Microb. Ecol. 79, 770–784. doi: 10.1007/s00248-019-01422-7 31432245

[B58] Suriyaphol P. Chiu J. K. H. Yimpring N. Tunsagool P. Mhuantong W. Chuanchuen R. . (2021). Dynamics of the fecal microbiome and antimicrobial resistome in commercial piglets during the weaning period. Sci. Rep. 11, 18091. doi: 10.1038/s41598-021-97586-9 34508122 PMC8433359

[B59] Techtmann S. M. Robb F. T. (2010). Archaeal-like chaperonins in bacteria. Proc. Natl. Acad. Sci. 107, 20269–20274. doi: 10.1073/pnas.1004783107 21057109 PMC2996707

[B60] Torres M. D. T. Wan F. de la Fuente-Nunez C. (2025). Deep learning reveals antibiotics in the archaeal proteome. Nat. Microbiol. 10, 2153–2167. doi: 10.1038/s41564-025-02061-0 40796684 PMC12408343

[B61] Uritskiy G. V. DiRuggiero J. Taylor J. (2018). MetaWRAP—a flexible pipeline for genome-resolved metagenomic data analysis. Microbiome 6, 158. doi: 10.1186/s40168-018-0541-1 30219103 PMC6138922

[B62] Volmer J. G. McRae H. Morrison M. (2023). The evolving role of methanogenic archaea in mammalian microbiomes. Front. Microbiol. 14. doi: 10.3389/fmicb.2023.1268451 37727289 PMC10506414

[B63] Wang Y. Wegener G. Williams T. A. Xie R. Hou J. Tian C. . (2021). A methylotrophic origin of methanogenesis and early divergence of anaerobic multicarbon alkane metabolism. Sci. Adv. 7 (27), eabj1453. doi: 10.1126/sciadv.abj1453 34215592 PMC11057702

[B64] Wickham H. (2016). Ggplot2: Elegant Graphics for Data Analysis. New York: Springer-Verlag.

[B65] Wickham H. Averick M. Bryan J. Chang W. McGowan L. François R. . (2019). Welcome to the tidyverse. J. Open Source Software 4, 1686. doi: 10.21105/joss.01686

[B66] Woese C. R. Fox G. E. (1977). Phylogenetic structure of the prokaryotic domain: The primary kingdoms. Proc. Natl. Acad. Sci. 74, 5088–5090. doi: 10.1073/pnas.74.11.5088 270744 PMC432104

[B67] Woese C. R. Kandler O. Wheelis M. L. (1990). Towards a natural system of organisms: proposal for the domains Archaea, Bacteria, and Eucarya. Proc. Natl. Acad. Sci. 87, 4576–4579. doi: 10.1073/pnas.87.12.4576 2112744 PMC54159

[B68] Wood D. E. Lu J. Langmead B. (2019). Improved metagenomic analysis with Kraken 2. Genome Biol. 20, 257. doi: 10.1186/s13059-019-1891-0 31779668 PMC6883579

[B69] Wu L.-Y. Wijesekara Y. Piedade G. J. Pappas N. Brussaard C. P. D. Dutilh B. E. (2024). Benchmarking bioinformatic virus identification tools using real-world metagenomic data across biomes. Genome Biol. 25, 97. doi: 10.1186/s13059-024-03236-4 38622738 PMC11020464

[B70] Xiong X. Rao Y. Tu X. Wang Z. Gong J. Yang Y. . (2022). Gut archaea associated with bacteria colonization and succession during piglet weaning transitions. BMC Vet. Res. 18, 243. doi: 10.1186/s12917-022-03330-4 35751084 PMC9229118

[B71] Xu Q. Qiao Q. Gao Y. Hou J. Hu M. Du Y. . (2021). Gut microbiota and their role in health and metabolic disease of dairy cow. Front. Nutr. 8. doi: 10.3389/fnut.2021.701511 34422882 PMC8371392

[B72] Yang J. Chen R. Peng Y. Chai J. Li Y. Deng F. (2023). The role of gut archaea in the pig gut microbiome: a mini-review. Front. Microbiol. 14. doi: 10.3389/fmicb.2023.1284603 37876779 PMC10593451

[B73] Yang B. Yang J. Chen R. Chai J. Wei X. Zhao J. . (2024). Metagenome-assembled genomes of pig fecal samples in nine European countries: Insights into antibiotic resistance genes and viruses. Microorganisms 12, 2409. doi: 10.3390/microorganisms12122409 39770612 PMC11676251

[B74] Zeineldin M. Megahed A. Blair B. Aldridge B. Lowe J. (2021). Metagenomic analysis of the fecal archaeome in suckling piglets following perinatal tulathromycin metaphylaxis. Animals 11, 1825. doi: 10.3390/ani11061825 34207278 PMC8235425

[B75] Zhang J. Li X. Klümper U. Lei H. Berendonk T. U. Guo F. . (2022). Deciphering chloramphenicol biotransformation mechanisms and microbial interactions via integrated multi-omics and cultivation-dependent approaches. Microbiome 10, 180. doi: 10.1186/s40168-022-01361-5 36280854 PMC9590159

[B76] Zhou H. He K. Chen J. Zhang X. (2022). LinDA: linear models for differential abundance analysis of microbiome compositional data. Genome Biol. 23, 95. doi: 10.1186/s13059-022-02655-5 35421994 PMC9012043

[B77] Zimmerman S. Innocenti A. Casini A. Ferry J. G. Scozzafava A. Supuran C. T. (2004). Carbonic anhydrase inhibitors. Inhibition of the prokariotic beta and gamma-class enzymes from Archaea with sulfonamides. Bioorg. Med. Chem. Lett. 14, 6001–6006. doi: 10.1016/j.bmcl.2004.09.085 15546717

