# Supplementary figures and images for: Dynamics of archaeal diversity and functionality in the piglet gut microbiome under common antimicrobial treatments
Source: Front Cell Infect Microbiol. 2026 Jul 16;16:1833734. doi: 10.3389/fcimb.2026.1833734 (PMC13422161; doi:10.3389/fcimb.2026.1833734)

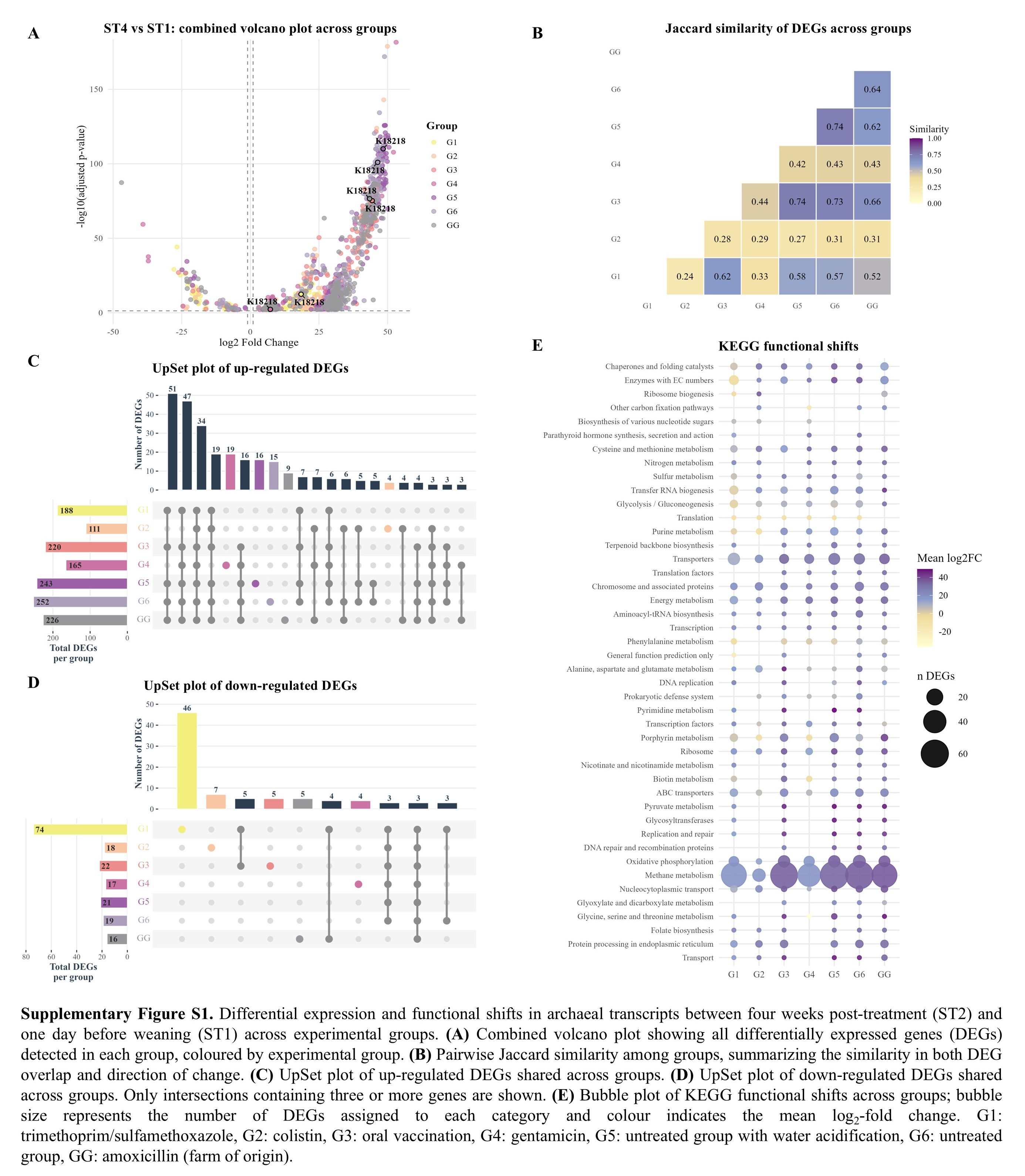

Supplement: Supplementary Figure 1 — Differential expression and functional shifts in archaeal transcripts between four weeks post-treatment (ST2) and one day before weaning (ST1) across experimental groups. (A) Combined volcano plot showing all differentially expressed genes (DEGs) detected in each group, coloured by experimental group. (B) Pairwise Jaccard similarity among groups, summarizing the similarity in both DEG overlap and direction of change. (C) UpSet plot of up-regulated DEGs shared across groups. (D) UpSet plot of down-regulated DEGs shared across groups. Only intersections containing three or more genes are shown. (E) Bubble plot of KEGG functional shifts across groups; bubble size represents the number of DEGs assigned to each category and colour indicates the mean log2-fold change. G1: trimethoprim/sulfamethoxazole, G2: colistin, G3: oral vaccination, G4: gentamicin, G5: untreated group with water acidification, G6: untreated group, GG: amoxicillin (farm of origin). [file Image1.jpeg]
